# Subcutaneous immunotherapy with depigmented-polymerized allergen extracts: a systematic review and meta-analysis

**DOI:** 10.1186/s13601-019-0268-5

**Published:** 2019-06-05

**Authors:** Ralph Mösges, Antonio Valero Santiago, Silke Allekotte, Nilufar Jahed, Anatoli Astvatsatourov, Angelika Sager, Jaime Sánchez-López

**Affiliations:** 1 CRI – Clinical Research International Ltd, Cologne, Germany; 2 Institute of Medical Statistics and Computational Biology, Faculty of Medicine, University of Cologne, Cologne, Germany; 3 Department of Pulmonology and Respiratory Allergy, Hospital Clinic Barcelona, Institut d’Investigacions Biomèdiques August Pi i Sunyer (IDIBAPS), Centro de Investigaciones Biomedicas en Red de Enfermedades Respiratorias (CIBERES), Universitat de Barcelona, Barcelona, Spain; 4 Clinical Trials Centre Cologne, Faculty of Medicine, University of Cologne, Cologne, Germany; 5 Laboratorios LETI, Gran Via de les Corts Catalanes 184, 08038 Barcelona, Spain

**Keywords:** Allergen immunotherapy, Allergic rhinoconjunctivitis, Bronchial asthma, Meta-analysis, Product-specific

## Abstract

**Background:**

Double-blind, placebo-controlled trials (DBPCTs) have confirmed the efficacy of allergen-specific immunotherapy (AIT) with depigmented-polymerized allergen extracts (DPAEs). This systematic review evaluates the efficacy of AIT using different allergens in different severity stages of rhinoconjunctivitis with or without asthma in the pollen studies and asthma and rhinitis in the house dust mite studies in comparison to placebo.

**Methods:**

We used MEDLINE, Embase, CENTRAL and LILACS databases to review DBPCTs published until July 2016. The combined symptom and medication score (cSMS) served as primary endpoint. The total rhinoconjunctivitis symptom score (RCSS) and total score in Rhinoconjunctivitis Quality of Life Questionnaire (RQLQ) were secondary efficacy endpoints. Solicited local and systemic adverse events were secondary safety endpoints. We assumed a random effects model with standardized mean differences (SMDs) or mean differences as summary statistics. In a subgroup analysis, we classified the studies following the GINA (Global Initiative for Asthma) and ARIA (Allergic Rhinitis and its Impact on Asthma) guidelines for rhinoconjunctivitis and asthma severity.

**Results:**

Six DBPCTs in pollen and 2 trials in house dust mites (HDM) were selected. Patients (N = 915) with intermittent or mild persistent asthma were included in 3 (37.5%) and 5 (62.5%) trials, respectively. Two (25%) HDM studies included patients with moderate persistent asthma, 4 trials patients with moderate-to-severe rhinoconjunctivitis. Treatment periods ranged from 12 to 24 months. AIT with DPAEs yielded significantly lower cSMS (SMD: 1.9, 95% CI: 0.9–2.8) and RQLQ (SMD: 0.3, 95% CI: 0.1–0.5) values than did placebo. An exploratory analysis of cSMS and RCSS suggested that the efficacy of AIT treatment with DPAEs was higher in trials including patients with more severe rhinoconjunctivitis and asthma. A publication bias was not detected. Heterogeneity between individual studies was explained by differences in severity. Patients receiving DPAEs did not experience a significantly higher risk of local (OR: 1.55, 95% CI: 0.86–2.79) or systemic reactions (OR: 1.94, 95% CI: 0.98–3.84).

**Conclusions:**

Compared to placebo, AIT with DPAEs is effective in patients with pollen- or HDM-induced rhinoconjunctivitis with or without allergic asthma and improves health-related quality of life. It does not differ significantly in safety and tolerability.

**Electronic supplementary material:**

The online version of this article (10.1186/s13601-019-0268-5) contains supplementary material, which is available to authorized users.

## Background

The only disease-modifying treatment for allergic rhinoconjunctivitis is allergen-specific immunotherapy (AIT) [1, 2] either by subcutaneous injection (SCIT) or by sublingual administration (SLIT). Numerous clinical trials and meta-analyses based on such studies have documented clinical efficacy for different kinds of pollen and house dust mite (HDM) extracts [3].

Given the heterogeneity in the evidence for different AIT products, it appears mandatory that each product should be evaluated individually [4].

Depigmented-polymerized allergen extracts (DPAEs) adsorbed onto aluminum have demonstrated their clinical effectiveness while keeping a low risk profile in several studies conducted with different allergens [5–7].

Because of the good safety profile, DPAEs can not only be used in conventional, but also in ultra-rush updosing schemes [8].Previous double-blind placebo-controlled trials (DBPCTs) have demonstrated the efficacy of AIT with DPAEs [9–11]. However, some studies included small sample sizes (< 100 patients) [12] and have limited validity so that it is not possible to translate their results to the allergic rhinoconjunctivitis patient population. Additionally, the efficacy of AIT with DPAEs has been studied in different severity stages of rhinoconjunctivitis and asthma [12–16]. None of the individual studies allow the proper comparison differences in the efficacy of DPAEs between rhinoconjunctivitis and asthma subgroups. A meta-analysis can combine results of several studies leading to a higher statistical power and more robust point estimate than is possible from the results derived from any individual study. Furthermore, this method is suitable for exploring the influence of study characteristics in the efficacy of the treatment evaluated [17].

A recent meta-analysis [3] has elegantly shown the efficacy and safety of AIT. However, mixing products with different characteristics has its limitations, as the results from one product must not necessarily be extrapolated to another. Therefore, we developed an AIT meta-analysis from the product perspective, including an efficacy analysis based on disease severity.

## Methods

### Search strategy

A comprehensive search for journal articles containing information on the treatment of allergic rhinoconjunctivitis and asthma up to July 2016 was carried out on Embase, MEDLINE, Cochrane Central Register of Controlled Trials (CENTRAL) in The Cochrane Library, and on the Latin American and Caribbean Literature on Health Sciences (LILACS). The search strategy retrieved citations from databases containing the subject heading “depigmented” and “polymerized” and was limited to controlled trials. In MEDLINE we limited the search to clinical trials using the systematic search algorithm proposed by Robinson and Dickersin [18] (see Additional file 1). The terms “polymerized” or “depigmented” did not find positive results in the LILACS database; therefore, the search strategy to retrieve citations in LILACS containing the subject “immunotherapy” was limited to controlled trials AND allergy AND immunology journals.

There were no language restrictions. All studies published up to 13 July 2016 were included in this systematic review.

The protocol has been published in the International prospective register of systematic reviews (PROSPERO) under the number CRD42016042866.

### Endpoints

The primary endpoint was a combined symptom medication score (cSMS) in DBPCTs with DPAEs analyzed at the time point and season (pollen studies) of the primary analysis in the original studies.

The secondary efficacy endpoints considered were health-related quality of life (HRQL) as measured by the total Rhinoconjunctivitis Quality of Life Questionnaire (RQLQ) score, cSMS and total rhinoconjunctivitis symptom score (RCSS) in all study seasons (12 and 24 months of treatment). For the assessment of safety, the occurrence of solicited local and systemic adverse events was considered. Systemic allergic reactions were classified and graded in the publications according to EAACI position papers of [19] and [20], respectively.

The covariates for the efficacy endpoints collected from each trial were severity of the treated allergic disease (rhinoconjunctivitis and asthma, ARIA and GINA classifications), treatment duration (months), duration of the evaluation period (months), number of subjects in the intention-to-treat (ITT) set, allergen studied (pollen and HDM), publication year, participant’s age (age groups classified according to PDCO criteria [21]), exclusion rate in the per protocol (PP) set, and the methodological quality score as evaluated using the Jadad scale [22].

#### Asthma and rhinoconjunctivitis severity subgroup

In order to evaluate the rhinoconjunctivitis severity, we grouped the studies in two groups, following the ARIA guidelines [23]:

- (0) Patients with allergic rhinoconjunctivitis (any severity was included),
- (1) Patients with moderate to severe allergic rhinoconjunctivitis (mild severity was excluded from these studies).

In order to evaluate asthma severity we grouped the studies in three groups, according to the GINA severity classification [24]:

- (0) Only patients with intermittent asthma,
- (1) Patients with intermittent to mild persistent asthma (moderate and severe asthma excluded),
- (2) Patients with intermittent to moderate persistent asthma (severe asthma excluded).

A combined score was then generated in order to classify the studies based on the severity of both rhinoconjunctivitis and asthma:

- (0) corresponded to studies including only patients with intermittent asthma patients – with any severity in rhinoconjunctivitis;
- (1) to studies including intermittent to persistent asthma and mild rhinoconjunctivitis;
- (2) to studies including intermittent to mild persistent asthma and moderate to severe rhinoconjunctivitis; and
- (3) to studies including intermittent to moderate persistent asthma and moderate to severe rhinoconjunctivitis.

For a detailed list regarding the distribution and severity of asthma among the analyzed studies, please refer to Additional file 2.

### Data extraction and analysis

The analyzed data were extracted from the published articles. The means and standard deviations of cSMS, RCSS and overall RQLQ score in active (immunotherapy with DPAEs) and placebo groups were extracted for each study. Unlike the RCSS and the medication score (MS), the cSMS was not reported in all the studies. In such cases, the method validated by Clark and Schall [25] was used to combine the RCSS and the MS. As daily scores were not described in the published articles, in accordance with properties of integral calculus, the total cSMS was approximated using the sum of the total symptom and medication scores from each publication. The agreement between the total cSMS as calculated with daily scores from clinical study reports (CSR) and as calculated with the total RCSS and MS from published papers was analyzed. The HRQL was only analyzed in studies implementing RQLQ measures. When the median and range were published, they were described as mean and standard deviation according to Hozo et al. [26]. The CSR were provided by the company for comparative purposes.

For the safety analysis, the number of patients who had developed local and systemic adverse events and the number of events of each trial was determined, regardless of the severity class of the adverse event.

Two independent reviewers identified and selected all publications reporting randomized DBPCTs of immunotherapy with DPAEs in allergic rhinoconjunctivitis and/or asthma. They assessed whether the publications met the criteria for inclusion. Each reviewer independently analyzed all included papers and recorded the relevant data concerning primary and secondary endpoints and the previously described relevant covariates on a predefined form.

The data recorded by the two reviewers were compared. When no agreement was found in a particular issue, the original paper was reanalyzed until a consensus decision was reached. Studies published as continuations of previously published studies were excluded to prevent repetition of data.

### Statistical analysis

Where clinically and statistically appropriate, meta-analyses were undertaken using random-effects modeling. Standardized mean difference (SMD) was used as summary statistics.

The agreement between the total cSMS as calculated using the daily RCSS and MS from CSR and as calculated with the total scores from published articles were evaluated using two-way intra-class correlation coefficients (ICCs), scatter plots and Bland–Altman plots. The normality assumption of score differences was assessed with the Shapiro test.

#### Heterogeneity analysis

Heterogeneity was identified with the Q test (p ≤ 0.1) [27]. Moreover the I^2^ index, which indicates the percentage of variance in a meta-analysis that is attributable to study heterogeneity, was used to quantify heterogeneity, considering as homogeneous studies with I^2^ values below 25%, low heterogeneity for values between 25 and 50%, moderate heterogeneity for values in the interval 50–75% and high heterogeneity for values over 75% [28].

#### Subgroup analysis

Subgroup analyses were conducted for study covariates with an explorative purpose. Meta-regression was used to examine the impact of covariates on study differences between placebo and active groups. In order to analyze the scores of the different pollen seasons described within the individual publications, these scores were included independently in the regression and the study identifier was analyzed as a random effect.

#### Publication bias

To handle publication bias, we used the fail-safe calculation, a simple procedure by which one can estimate whether publication bias (if it exists) may be safely ignored. A fail-safe N indicates the number of insignificant, unpublished (or missing) studies that would need to be added to a meta-analysis to reduce an overall statistically significant result to insignificance. If the fail-safe N is higher than the number of observed trials (8 studies), we can assume that there is no relevant publication bias. The fail-safe N values were computed in accordance with the Rosenberg approach [29]. Moreover, Egger’s test method based on the funnel plot was used to evaluate meta-analysis bias. With a *p* value of > 0.1, Egger’s regression test suggests the absence of publication and unaccounted heterogeneity bias, as the symmetric funnel plot reflects. Egger’s test estimations are not calculated with less than 3 studies [17].

The results were presented in forest plots and summary tables. For the study weights, the inverse variance method was applied. The level of significance for differences between AIT with DPAEs in meta-analysis was ≤ 0.05. The level of significance for detecting bias with heterogeneity Q and Egger’s test was ≤ 0.1, because these tests may be underpowered with few studies. Finally, due to the exploratory purpose of the meta-regression analysis, the level of significance was ≤ 0.1. All analyses were performed using R v3.0.2 software (The R Foundation for Statistical Computing, Vienna, Austria) and library “metafor” v2016-09-25 [29].

## Results

### Systematic review

**Fig. 1:**
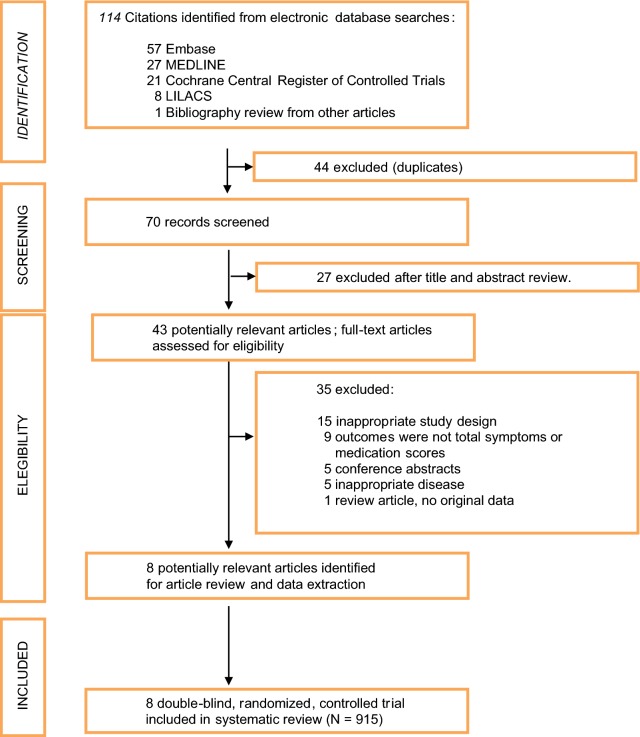
Flow Diagram of depigmented polymerized immunotherapy studies selection. Data recorded by two researchers were compared. When no agreement was found in a particular issue, the original paper was reanalyzed until a consensus decision was reached. Studies published as continuations of previously published studies were excluded to prevent repetition of data

From 114 citations, a total of 43 studies were initially selected for systematic review (excluding duplicates after the review of titles and abstract). Finally, 8 publications (N = 915 patients) fulfilled the study selection criteria (Fig. 1, Additional File 3).

For half of the studies, the primary efficacy analysis was performed using data from the first year, which was the season for the primary outcome of AIT: Colás [13], Alvarez-Cuesta [15], Ameal [14], Garcia-Robaina [16], and for the other half of the studies using data from the second year of treatment (being the season for the primary outcome): Pfaar [9–11], Höiby [12], depending on the respective definition of the primary analysis in the trials. The secondary efficacy endpoints considered the previous evaluation periods for cSMS and RCSS and included the first year of AIT with DPAEs in Pfaar [10] and [11]. Four studies reported total RQLQ scores and were included in the HRQL analysis (N = 692 patients).

For the secondary safety endpoints, only those studies were considered that described the corresponding data. Therefore, the number of patients differs between the different evaluations.

The number of patients developing local reactions [Alvarez-Cuesta (2005), Colás (2006), Höiby (2010), Pfaar (2010), Pfaar (2012), Pfaar (2013)] and the number of patients developing systemic reactions [9–12, 14, 16] after SCIT with DPAEs were reported in 6 studies, respectively (local: N = 838; systemic N = 836). Additionally, only the number of systemic reactions was reported and included in 6 trials [N = 835; 10–14, 16].

### Study characteristics

The studies were published from 2005 to 2013. Patients were treated for allergy to birch pollen (3 studies), grass pollen (3 studies), HDM (2 studies), Russian thistle pollen (1 study) and olive pollen (1 study), including mixtures of different, of the abovementioned, allergens. Patients with intermittent asthma were included in 3 (37.5%) DBPCTs and those with mild asthma in 5 (62.5%) DBPCTs. Patients with moderate asthma were included in the 2 (25%) HDM studies. Four DBPCTs (50%) included patients with moderate to severe rhinoconjunctivitis. All studies had a treatment period of at least one year. The baseline characteristics are summarized in Table 1.

**Table 1:** Summary of articles and patient characteristics

Study characteristics	N = 8 DBPCTs; 915 patients
Publication year, range	(2005 to 2013)
Multicenter vs. monocenter	4 (50%) vs. 4 (50%)
Allergen used (pollen vs. house dust mites)	6 (75%) vs. 2 (25%)
Treatment duration, range (months)	(12 to 24)
Duration of symptoms and medication evaluation period, range (months)	(1 to 12)
Total score in Jadad methodology scale, range	(2 to 5)
Number of patients per study, range	45 to 269
Total number of patients in active group, n (%)	591 (64.6%)
Placebo group, n (%)	324 (35.4%)
Patient’ s age, mean (range), years	32 (7 to 69)
PDCO groups of age in included studies	
Children and adolescents included, n (%)	2 (25%)
Adolescents included, n (%)	4 (50%)
Only adults included, n (%)	2 (25%)
Rhinoconjunctivitis severity in included studies	
Any severity included, n (%)	4 (50%)
Only moderate to severe, n (%)	4 (50%)
Asthma severity in included studies	
Only include intermittent asthma, n (%)	3 (37.5%)
Intermittent to mild persistent included, n (%)	3 (37.5%)
Intermittent to moderate persistent included, n (%)	2 (25%)
% pts not included in per protocol set: active group	(5% to 24%)
Placebo group	(5% to 25%)
Studies with HRQL evaluated with RQLQ	
Yes* (692 patients)	4 (50%)
No**	4 (50%)

*HRQL* health-related quality of life; n: number of patients, *PDCO* European Medicines Agency. Paediatric Committee, *DBPCT* double-blind placebo-controlled trial, *QoL* Quality of Life

*Pfaar (2013)[11], Pfaar (2012)[10], Pfaar (2010)[9], Álvarez-Cuesta (2005)[15]

**Höiby (2010)[12], García-Robaina (2006)[16], Ameal (2005)[14] and Colás (2006). It is interesting to note that the Höiby (2010) study evaluated health-related QoL with combined sum of scores of the pediatric RQLQ and RQLQ. This study was not evaluated in QoL analysis because they did not report RQLQ scores, separately

The information extracted from original articles is summarized in Tables 2 and 3.

**Table 2:** Main methodological characteristics of included articles

No.	Year	First author	Duration (months)		Sites	Allergen	Scores in Jadad scale			
			Treatment period	Evaluation Period			Randomization	Blind	Drop-outs	Total
1	2010	Pfaar	12/24	4	Multicenter	Birch pollen	1	2	1	4
2	2013	Pfaar	12/24	7	Multicenter	Birch/grass	2	2	1	5
3	2012	Pfaar	12/24	4.5	Multicenter	Grass	1	2	1	4
4	2010	Höiby	12/24	1	Multicenter	Birch	2	2	1	5
5	2006	Colás	12	1	Monocenter	Russian thistle	1	1	1	3
6	2005	Ameal	12	1	Monocenter	House dust mite	1	1	1	3
7	2005	Álvarez-Cuesta	12	1	Monocenter	Grass/olive	1	1	0	2
8	2006	García-Robaina	12	12	Monocenter	House dust mite	1	1	1	3

**Table 3:** Main clinical characteristics of included articles

No.	Year	First autor	Rhinoconjunctivitis severity (ARIA)	Asthma severity (GINA)	Mean age (SD or range)	PDCO groups of age	Patients not included in PP analysis	
							Active group (%)	Placebo group (%)
1	2010	Pfaar	Mild to severe	Only intermittent	39 (18 to 65)	Only adults	14.6	14.9
2	2013	Pfaar	Moderate to severe	Only intermittent	31 (12.4)	Adolescent included	14.5	17.2
3	2012	Pfaar	Moderate to severe	Only intermittent	33 (11 to 69)	Children Included	23.7	25
4	2010	Höiby	Mild to severe	Intermittent to mild	33 (7 to 69)	Children Included	22.6	30
5	2006	Colás	Mild to severe	Intermittent to mild	34 (18 to 51)	Only adults	5	5
6	2005	Ameal	Mild to severe	Intermittent to moderate	23 (14 to 48)	Adolescent included	9.4	16.1
7	2006	García-Robaina	Moderate to severe	Intermittent to moderate	24 (9.3)	Adolescent included	20	20
8	2005	Álvarez-Cuesta	Moderate to severe	Intermittent to mild	28 (17 to 58)	Adolescent included	–	–

*ARIA* allergic rhinoconjunctivitis and its impact on Asthma, *GINA* global initiative for Asthma, *PDCO* European Medicines Agency. Paediatric Committee, *PP* Per protocol, *SD* Standard deviation

### Primary efficacy analysis

**Fig. 2:**
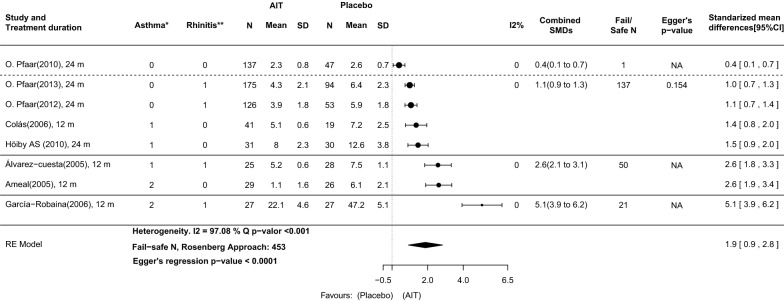
AIT with DPAEs compared with placebo at the time of primary analysis in original publications. Outcome: combined symptom and medication score (cSMS) calculated with total scores from published articles. Four subgroups of asthma and rhinoconjunctivitis severity are delimited by solid and dashed lines. *Asthma classification: (0) Only intermittent asthma patients included; (1) Intermittent to mild persistent asthma patients included; (2) Intermittent to moderate persistent asthma patients included. **Rhinoconjunctivitis classification: (0) Any rhinoconjunctivitis severity included; (1) Moderate to severe rhinoconjunctivitis included. 95%CI: 95% confidence interval; *m* months, *SD* standard deviation, *SMDs* standardized mean difference

The analysis of the cSMS at the time of the primary analysis showed a SMD of 1.9 (95% CI: 0.9–2.8), suggesting a strong effect in favor of DPAEs (Fig. 2). Although a remarkable degree of heterogeneity between the results of individual studies was reported (I^2^ = 97.1%, Q test and Egger’s test p-values ≤ 0.001) the fail-safe number was 453, high enough to confirm the robustness of the results against publication bias.

**Fig. 3:**
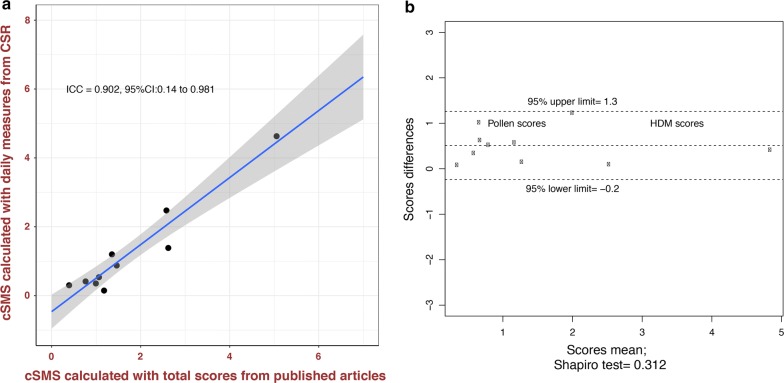
Agreement between cSMS calculated with daily measures from CSR and calculated with total scores in a Scatter plot (**a**) and Bland–Altman plot (**b**). From published articles in all study seasons [9–16]. **a** The points represent the intersection between the two cSMS values (cSMS calculated with daily measures from the CSR and calculated with total scores from published articles). Strong agreement can be observed between the two measurements (ICC: 0.902, 95% CI: 0.14–0.981). **b** The X-axis represents the sum of two cSMS values divided by two; the Y-axis represents the differences between the two measures of cSMS. Strong agreement between the two measurements can be seen because there are no points outside the 95% limits of agreement (dashed lines). It is common to compute 95% limits of agreement for each comparison (average difference ± 1.96 standard deviation of the difference), which tell us how far apart measurements observed using two methods were more likely to be for different cases. Contrary to expectations, in the articles that published cSMS the two measures are not equal. There are slight differences between cSMS values extracted from clinical study reports (CSRs) and those extracted from publications. This is due to the fact that in the published articles provided only the median and range, and therefore they should be transformed to mean and standard deviation. Whereas in the CSRs the mean and the standard deviation were available. *cSMS* combined symptoms and medication score, *CSR* clinical study report, *HDM* house dust mite, *ICC* intra-class correlation coefficient

**Fig. 4:**
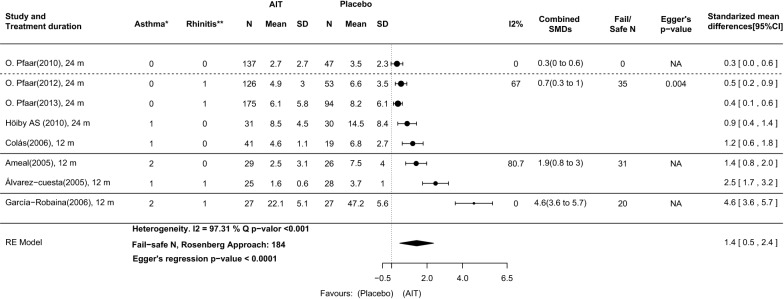
AIT with DPAEs compared with placebo at the time of primary analysis in original studies. Outcome: combined symptom and medication score (cSMS) calculated with daily measures from CSR. Four subgroups of asthma and rhinoconjunctivitis severity are delimited by solid and dashed lines. *Asthma classification: (0) Only intermittent asthma patients included; (1) Intermittent to mild persistent asthma patients included; (2) Intermittent to moderate persistent asthma patients included. **Rhinoconjunctivitis classification: (0) Any rhinoconjunctivitis severity included; (1) Moderate to severe rhinoconjunctivitis included. 95%CI: 95% confidence interval; *m* months, *SD* standard deviation, *SMDs* standardized mean differences

The agreement between the total cSMS calculated using the daily RCSS and MS and the cSMS calculated using the total scores from published articles was high (ICC: 0.902, 95% CI: 0.14–0.981; 95% CI Bland–Altman boundaries were not crossed) (Fig. 3). This high agreement suggests that cSMS results calculated with a total score from published articles show equivalent results to cSMS values calculated with daily measures from CSRs. In accordance, we observed that patients treated in the DPAEs group showed a significantly lower cSMS than did those receiving placebo (SMD: 1.4, 95% CI: 0.5–2.4) when daily measures from CSRs were used. The cSMS values in AIT with DPAEs were significantly lower than those in placebo in all studies compared (Fig. 4).

**Fig. 5:**
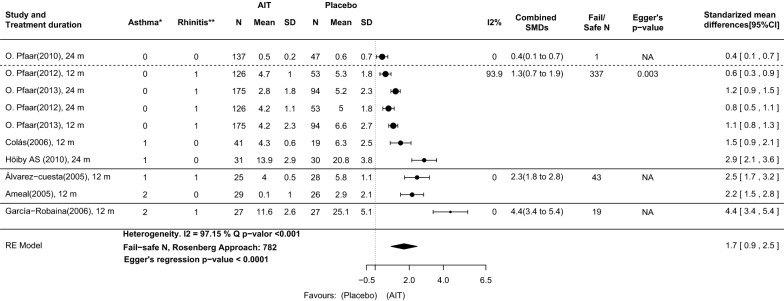
Rhinoconjunctivitis symptom score (RCSS) for AIT with DPAEs and for placebo in all study seasons. Four subgroups of asthma and rhinoconjunctivitis severity are delimited by solid and dashed lines. *Asthma classification: (0) Only intermittent asthma patients included; (1) Intermittent to mild persistent asthma patients included; (2) Intermittent to moderate persistent asthma patients included. **Rhinoconjunctivitis classification: (0) Any rhinoconjunctivitis severity included; (1) Moderate to severe rhinoconjunctivitis included. 95%CI: 95% confidence interval; *m* months, *RCSS* rhinoconjunctivitis symptom score, *SD* standard deviation, *SMDs* standardized mean differences

Additionally, the analysis of the RCSS evaluated in all study seasons showed equivalent results. The patients undergoing AIT with DPAEs had a significantly lower RCSS than those receiving placebo (SMD: 1.7, 95% CI: 0.9–2.5) and the RCSS values for AIT with DPAEs were significantly lower than those for placebo in all studies compared (Fig. 5).

### Secondary efficacy analysis

#### RQLQ evaluated in all study seasons

**Fig. 6:**
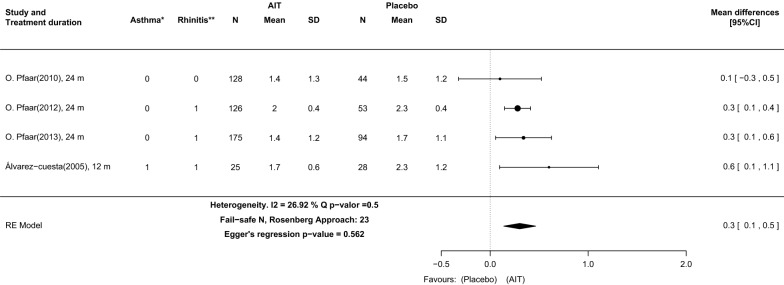
DPAEs versus placebo in all pollen seasons of original studies. Outcome: health-related quality of life (HRQL) measured with Rhinoconjunctivitis Quality of Life Questionnaire (RQLQ). *Asthma classification: (0) Only intermittent asthma patients included; (1) Intermittent to mild persistent asthma patients included; (2) Intermittent to moderate persistent asthma patients included. **Rhinoconjunctivitis classification: (0) Any rhinoconjunctivitis severity included; (1) Moderate to severe rhinoconjunctivitis included. 95%CI: 95% confidence interval; *m* months, *SD* standard deviation

The RQLQ total scores were significantly higher in the group undergoing AIT with DPAEs than in the placebo group (0.3, 95% CI: 0.1–0.5). The I^2^ index, the fail-safe N and Egger’s test results indicate that there was neither significant heterogeneity (I^2^ = 26.9, p = 0.5) nor publication bias (Fail-safe N = 23, p = 0.58) between study subgroups (Fig. 6).

#### Exploratory analysis of the efficacy of AIT with DPAEs vs. placebo depending on severity stages of rhinoconjunctivitis and asthma and type of allergen

##### cSMS at the season of primary analysis in original studies (8 measures from 8 publications)

The standardized mean difference in the cSMS between AIT with DPAEs and placebo in the four severity scores were: 0.4, (95%-CI: 0.1–0.7), 1.1 (95% CI: 0.9–1.3), 2.6 (95% CI: 2.1–3.1) and 5.1 (95% CI: 3.9–6.2) for scores 0, 1, 2 and 3, respectively (Fig. 2). These results suggest that for AIT with DPAEs the efficacy gain as compared with that for placebo grows proportionally to the asthma and rhinoconjunctivitis severity of the patients included (Figs. 2 and 6). There was not significant (p > 0.1) or relevant (I^2^ < 25%) degree of heterogeneity between studies grouped in these categories.

Additionally, the analysis of the total cSMS as calculated with daily measures from the CSR reflects equivalent results. Although the difference in means calculated with this method is lower in some studies, the SMD in the four severity categories grows proportionally to the severity of asthma and rhinoconjunctivitis in the patients included (Fig. 4).

##### cSMS evaluated in all study seasons (10 measures from 8 publications)

Considering the measures of all study seasons, the results are in accordance with previous analyses. The cSMS standardized mean differences between AIT with DPAEs and placebo in the four severity scores were: 0.4, (95% CI: 0.1–0.7), 1.1 (95% CI: 0.9–1.2), 2.6 (95% CI: 2.1–3.1) and 5.1 (95% CI: 3.9–6.2) for scores 0, 1, 2 and 3, respectively. Additionally, no significant (p > 0.1) or relevant (I^2^ < 25%) degree of heterogeneity were observed between the studies belonging to these subgroups (Table 4).

**Table 4:** Secondary analysis: cSMS and RCSS of AIT with DPAEs compared with placebo (all study seasons)

Study subgroups	GINA + ARIA score	Study subgroups	Efficacy evaluation period	cSMS as primary	All cSMS	All RCSS
Intermittent asthma Any rhinoconjunctivitis severity	0	Pfaar, 2010 (Pollen)	24 mo. (Primary)	0.4 (0.1 to 0.7)	0.4 (0.1 to 0.7)	0.4 (0.1 to 0.7)
Intermittent to mild asthma Moderate to severe rhinoconjunctivitis	1	Pfaar, 2012 (Pollen)	24 mo. (Primary) 12 mo. (Secondary)	1.1 (0.9 to 1.3)	1.1 (0.9 to 1.2)	1.3 (0.7 to 1.9)
		Pfaar, 2013 (Pollen)	24 mo. (Primary) 12 mo. (Secondary)			
		Colás, 2006 (Pollen)	12 mo. (Primary)			
		Höiby, 2010 (Pollen)	24 mo. (Primary)			
Intermittent to moderate asthma or Moderate to severe rhinoconjunctivitis	2	Alvarez-Cuesta, 2005 (Pollen)	12 mo. (Primary)	2.6 (2.1 to 3.1)	2.6 (2.1 to 3.1)	2.3 (1.8 to 2.8)
		Ameal, 2005 (HDM)	12 mo. (Primary)			
Intermittent to moderate asthma & Moderate to severe rhinoconjunctivitis	3	García-Robaina, 2006 (HDM)	12 mo. (Primary)	5.1 (3.9 to 6.2)	5.1 (3.9 to 6.2)	4.4 (3.4to 5.4)

*95% CI* 95% confidence interval, *AIT* allergen specific immunotherapy, *ARIA* Allergic Rhinitis and its Impact on Asthma, *cSMS* combined symptom and medication score, *GINA* Global Initiative for Asthma, *HDM* house dust mite, *mo* months, *RCSS* rhinoconjunctivitis symptom score, *SD* standard deviation, *SMD* standardized mean differences

##### RCSS evaluated in all study seasons (10 measures from 8 publications)

The RCSS standardized mean differences between AIT with DPAEs and placebo in the four severity scores were: 0.4, (95% CI: 0.1–0.7), 1.3 (95% CI: 0.7–1.9), 2.3 (95% CI: 2.8–2.8) and 4.4 (95% CI: 3.4–5.4) for scores 0, 1, 2 and 3, respectively. Contrary to what was observed in the analysis of the cSMS, we observed a high amount of heterogeneity (97, p < 0.001) in studies classified in these groups (Fig. 5).

##### Meta-regression

The meta-regression analysis concurs the previous results (Table 5). The analysis of the primary endpoint with meta-regression shows that efficacy of AIT with DPAEs was higher in trials that had included patients with more severe asthma or rhinoconjunctivitis, and in trials having a longer evaluation period and a larger control group sample size. These factors accounted for 100% of heterogeneity (I^2^ = 0, Q test p-value = 0.469). However, the number of studies is low (8 in number) in comparison with the number of predictors (6 in number), suggesting that the model is over-fitted and results cannot be correctly translated to other studies. To overcome this issue, we included in the meta-regression analysis the scores of all pollen seasons from the studies that had evaluated treatment efficacy after one or two years of treatment. Analyses of cSMS results suggest that the most important moderators associated with the higher efficacy of AIT with DPAEs than of placebo are rhinoconjunctivitis and asthma (Table 5). Moreover, we observed that the efficacy gain of AIT with DPAEs over placebo increases with increased treatment duration (p < 0.1) (Table 5). Finally, when the RCSS score was considered (Table 5), rhinoconjunctivitis (p = 0.1) and asthma severity were again the factors associated with a higher gain in efficacy of the AIT with DPAEs compared to placebo (Table 5). These results suggest that the efficacy of AIT with DPAEs improves with increasing rhinitis severity of the patients included in comparison to *placebo.*

**Table 5:** Meta-regression results

	Combined symptom and medication scores (cSMS)				Rhinoconjunctivitis symptom scores (RCSS)	
Meta-regression	At time of primary analysis in original studies (one measure by study)		All pollen seasons included (one measure by pollen season)		All pollen seasons included (one measure by pollen season)	
Higher difference between depigmented-polymerized allergen extracts and placebo	Mean difference 95%CI	p-value	Mean difference 95%CI	p-value	Mean difference 95%CI	p-value
*Asthma score*						
Intermittent to moderate included (0)	Reference category		Reference category		Reference category	
Mild to moderate included (1)	1.4 (0.8 to 1.9)	*< 0.001*	1.7 (1 to 2.4)	*< 0.001*	2.4 (0.3 to 4.5)	*< 0.001*
Only moderate included (2)	2.5 (1.7 to 3.2)	*< 0.001*	2.9 (2.2 to 3.7)	*< 0.001*	2.5 (0.3 to 4.6)	*< 0.001*
*Rhinoconjunctivitis*						
Mild excluded against included	0.8 (0.4 to 1.2)	*< 0.001*	0.7 (0.08 to 1.3)	*< 0.001*	0.9 (− 0.2 to 2)	*0.1*
*Durations of evaluation period*						
Increasing 1 month	0.1 (0.01 to 0.3)	*< 0.05*	0.1 (− 0.01 to 0.3)	*< 0.1*	Not Significant	
*Control group sample size*						
Increasing 10 patients	− 0.1 (− 0.2 to 0.0)	*< 0.05*	Not significant		Not Significant	
Heterogeneity Q test:	I^2^ = 0% p-value = 0.469		I^2^ = 18.3% p-value = 0.13		I^2^ = 84.15% p-value = 0.003	

Statistically significant values are in italics (p ≤ 0.1)

##### Type of allergen analysis

Furthermore, patients with more severe asthma were included in the 2 HDM studies. When the type of allergen (pollen or HDM) and not the severity of rhinoconjunctivitis and asthma are considered in the meta-regression analysis, HDM studies reported significantly lower cSMS (SMD: 2.4, 95% CI: 0.9–3.9, p = 0.001) and RCSS (SMD: 1.7, 95% CI: 0.01–3.6, p = 0.05) values compared with placebo than those presented in pollen studies.

### Secondary safety analysis

**Fig. 7:**
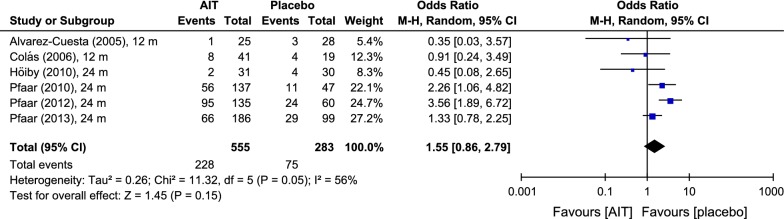
Odds ratios for local reactions under AIT with DPAEs and placebo. Data extracted from the original studies. Outcome: number of patients developing local reactions after immunotherapy administration. *AIT* allergen-specific immunotherapy, *CI* confidence interval, *m* months

The analysis of the number of patients developing at least one local reaction after administration of DPAEs showed an odds ratio of 1.55 [0.86; 2.79], based upon approximately 41% under active treatment versus 27% of patients taking placebo reporting at least one local reaction (Fig. 7).

**Fig. 8:**
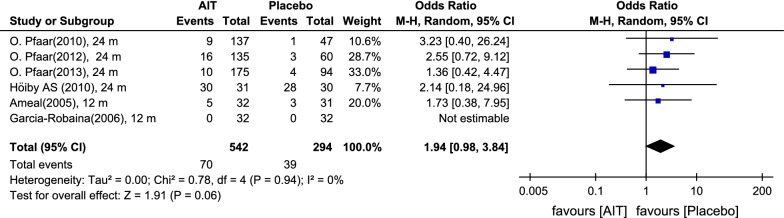
Odds rations for systemic reactions under AIT with DPAEs and placebo. Data extracted from the original studies. Outcome: number of patients developing systemic reactions after immunotherapy administration. *AIT* allergen-specific immunotherapy, *CI* confidence interval, *m* months

For the number of patients developing a systemic reaction, the odds ratio was 1.94 [0.98; 3.84], suggesting that almost twice as many patients receiving depigmented-polymerized allergen extract than those taking placebo developed a systemic reaction (Fig. 8). However, this difference was not quite significant.

**Fig. 9:**
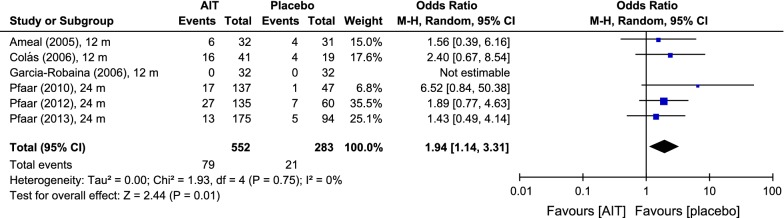
Risk ratios for systemic reactions under AIT with DPAEs and placebo. Data extracted from the original studies. Outcome: number of systemic reactions after immunotherapy administration. *AIT* allergen-specific immunotherapy, *CI* confidence interval, *m* months

The odds ratios of the numbers of patients developing adverse events are not statistically significant, but for the number of systemic reactions developed after the administration of DPAEs, the odds ratio achieved statistical significance (1.94 [1.14; 3.31 (p < 0.05)], Fig. 9).

## Discussion

The present meta-analysis demonstrates that AIT with DPAEs improves allergic symptoms, medication score and QoL in patients with rhinoconjunctivitis with or without allergic asthma sensitized to pollen or house dust mites. These results confirm the conclusions from previous positive DBPCTs [9–16]; and they are in agreement with the results found for subcutaneous immunotherapy in the largest immunotherapy meta-analysis done to date [3].

It may be regarded as a limitation of this product-line specific approach that no differentiation per allergen was made [4]. However, it was our intention to assess this individual form of treatment with identical production process and defined protocol of application. We have shown that the heterogeneity of the results has its origin in disease severity rather than in the allergen spectrum, which speaks for the identity of product specification.

The combined symptom medication score (cSMS) used in this analysis is not identical to the Combined Symptom and Medication Score (CSMS) recommended in the EAACI position paper [30], since the scoring of symptoms and medication used in the original publications was not in line with the classification of Pfaar et al. published later in [30].

As in previous publications [11, 12, 15], the results suggest that AIT with DPAEs significantly improves HRQL when compared to placebo. Analysis of HRQL could only be performed for 4 studies, including 75% of all patients (N = 692). However, the heterogeneity (26.9%) between studies and risk of publication bias (Fail-safe N = 23, Egger’s p = 0.5) was low. Therefore, similar results are expected for further DBPCTs that have not yet been conducted.

It is recommended that a primary efficacy endpoint in AIT trials should reflect both symptom severity as well as the intake of rescue medication [30]. In accordance, different publications and consensus papers have highlighted the advantages of using a combined symptom and medication score as a primary endpoint in allergy clinical trials [25, 30, 31]. Studies emphasized their advantages: Firstly to allow direct comparisons between different clinical trials and secondly to be associated with a large effect size when RCSS and MS are equally combined [25]. These characteristics make the cSMS a very interesting endpoint in the meta-analysis of clinical trials. One of the limitations of our approach based on published literature data instead of original data is that the new definitions of efficacy of the symptom medication score [30] and the safety and tolerability [32] could not be applied since the available data were not classified accordingly. However, cSMS is not usually considered in meta-analyses or it is not analyzed for all available studies [3, 28, 33–35], because it was not calculated in former publications, which displayed RCSS and MS independently. Our study suggests that the sum of total symptom and medication scores from each publication may be a valid method to approximate cSMS when scores are not calculated. Furthermore, cSMS was found to be more useful than RCSS to explain heterogeneity in rhinitis and asthma studies, when rhinoconjunctivitis and asthma severity are considered.

Despite focusing on rhinitis in the analysis presented here, results of exploratory heterogeneity analysis suggest that the efficacy of AIT with DPAEs against placebo grows proportionally to the rhinoconjunctivitis and asthma severity, which is in accordance with previous data [36, 37]. However, the external validity of this result is limited because there were only two studies with moderate asthma included in this analysis. Furthermore, asthma improvement was not documented in the majority of the studies and improvement of asthma symptoms could not be assessed. Moreover, no severe asthma patients were included in any study in accordance with previous regulatory authorities advice restricting asthma severity to mild and moderate in clinical trials [38]. There is a lack of evidence supporting or rejecting the use of immunotherapy in the severe asthma subgroup, and as it is emphasized by the regulatory guidelines, it is time to perform studies with this type of patients [38]. As shown in our safety and tolerability analysis, AIT with DPAEs has a low risk profile and the present study shows an improvement in efficacy in the persistent asthma compared to the intermittent asthma groups. Therefore, AIT with DPAEs could be a good candidate to test immunotherapy efficacy in patients with severe allergic asthma, given the fact that no anaphylaxis grade 3 or higher was observed in any of the studies.

As observed in previous studies [39, 40], patients with most severe asthma symptoms were allergic to a perennial allergen (house dust mite). The results suggest that superiority of AIT with DPAEs versus placebo is higher in perennial allergens than in seasonal allergens (birch or grass pollen).

Patient’s clinical characteristics in systematic reviews and meta-analyses are usually controlled by restricting the analysis to a particular allergen, in which the severity of symptoms between different studies are usually equivalent [35]; or are not considering rhinoconjunctivitis and asthma severity because it is not homogeneously described in the included studies [28]. The obtained results suggest that treatment differences between allergens may be properly analyzed with meta-analysis; and rhinoconjunctivitis and asthma severity are important factors to explain heterogeneity between studies.

## Conclusions

The present study demonstrates that AIT with DPAEs improves the allergic symptoms in patients with rhinoconjunctivitis with or without allergic asthma sensitized to pollen or house dust mites. Furthermore, HRQL was also better in AIT with DPAEs treatment then in placebo.

As an exploratory result, the meta-analysis suggests that AIT with DPAEs efficacy against placebo grows proportionally to the rhinoconjunctivitis and asthma severity. This finding should be considered in the design of future clinical trials and pharmacoeconomic reviews in order to confirm its usefulness.

## Additional files

**Additional file 1.** Search syntax in electronic databases. Search syntax in electronic databases a) in Embase, b) in PubMed, c) in Cochrane, d) in LILACS.

**Additional file 2.** Distribution and severity of asthma among the analyzed studies. A tabular overview of the number of included asthmatic patients in the analyzed studies and the grade of severity at the screening visit.

**Additional file 3.** Articles selected for systematic review. Tabular display of all articles selected for systematic review. Articles selected for analysis are highlighted in bold. The following sources were used: Embase (*1), MEDLINE (*2), Cochrane (*3), LILACS (*4) and BIBLIOGRAPHY REVIEW FROM OTHER SELECTED ARTICLES (*5).

## Data Availability

Not applicable.

## Abbreviations

AIT: allergen-specific immunotherapy
ARIA: Allergic Rhinitis and its Impact on Asthma
cSMS: combined symptom and medication score
CSR: clinical study report
DPAE: depigmented-polymerized allergen extract
DBPCT: double-blind, placebo-controlled trial
GINA: Global Initiative for Asthma
HDM: house dust mite
HRQL: health-related quality of life
ICC: intra-class correlation coefficient
ITT: intention to treat
MD: mean difference
MS: medication score
No: Number
PDCO: European Medicines Agency. Peadiatric Committee
PP: per protocol
QoL: Quality of Life
RCSS: rhinoconjunctivitis symptom score
Pt: Patients
RQLQ: Rhinoconjunctivitis Quality of Life Questionnaire
SCIT: subcutaneous immunotherapy
SLIT: sublingual immunotherapy
SMD: standardized mean difference
