# Supplementary material for: Subcutaneous immunotherapy with depigmented-polymerized allergen extracts: a systematic review and meta-analysis
Source: Clin Transl Allergy. 2019 Jun 5;9:29. doi: 10.1186/s13601-019-0268-5 (PMC6549305; doi:10.1186/s13601-019-0268-5)
Supplement: Supplementary file 1 — Additional file 1. Search syntax in electronic databases. Search syntax in electronic databases a) in Embase, b) in PubMed, c) in Cochrane, d) in LILACS. [file 13601_2019_268_MOESM1_ESM.docx]

**Additional file 1. Search syntax in electronic databases.**

| 1. **Search syntax in Embase:** |
| --- |
| depigmented AND polymerized |
| 1. **Search syntax in PubMed database:** |
| “depigmented [all] AND (polymerized [all]) AND ((randomized controlled trial [pt] OR controlled clinical trial [pt]) OR randomized controlled trial [mh] OR random allocation [mh] OR double-blind method [mh] OR single-blind method [mh] OR clinical trial [pt] OR clinical trials [mh] OR (“clinical trial” [tw]) OR ((singl* [tw] OR doubl* [tw] OR trebl* [tw] OR tripl* [tw]) AND (mask* [tw] OR blind* [tw])) OR (“latin Square” [tw]) OR placebos [mh] OR placebo* [tw] OR random* [tw] OR research design [mh:noexp] OR comparative study [all] OR evaluation studies [all] OR follow-up studies [mh] OR prospective Studies [mh] OR cross-over studies [mh] OR control* [tw] OR prospectiv* [tw] OR volunteer* [tw]) NOT (animal [mh] NOT human [mh])) “ |
|  |
| 1. **Search syntax in Cochrane database:** |
| “depigmented AND polymerized AND ((randomized controlled trial OR controlled clinical trial OR randomized controlled trial OR random allocation OR double-blind method **OR** single-blind method OR clinical trial OR clinical trials OR research design OR comparative study OR evaluation studies OR follow-up studies OR prospective studies) NOT (animal NOT human))” |
|  |
| 1. **Search syntax in Latin American and Caribbean Literature on Health Sciences (LILACS) database:** |
| tw:(immunotherapy) AND db:("LILACS") AND type_of_study:("clinical_trials") AND jd:("ALERGIA E INMUNOLOGIA") |

Search syntax in electronic databases a) in Embase b) in PubMed c) in Cochrane d) in LILACS.
