# Supplementary material for: Subcutaneous immunotherapy with depigmented-polymerized allergen extracts: a systematic review and meta-analysis
Source: Clin Transl Allergy. 2019 Jun 5;9:29. doi: 10.1186/s13601-019-0268-5 (PMC6549305; doi:10.1186/s13601-019-0268-5)
Supplement: Supplementary file 2 — Additional file 2. Distribution and severity of asthma among the analyzed studies. A tabular overview of the number of included asthmatic patients in the analyzed studies and the grade of severity at the screening visit. [file 13601_2019_268_MOESM2_ESM.docx]

**Additional File 2: Distribution and severity of asthma in the included studies**

| **Study** | | **Placebo** | | **Immunotherapy** | |
| --- | --- | --- | --- | --- | --- |
|  |  | **N total** | **N asthma (severity)** | **N total** | **N asthma (severity)** |
| Alvarez-Cuesta | Pollen | 28 | 3 (mild) | 25 | 2 (mild) |
| Höiby | Pollen | 30 | 12 (unknown) | 31 | 11 (unknown) |
| Pfaar 2010 | Pollen | 47 | 23 | 137 | 61 |
| Pfaar 2012 | Pollen | 53 | 1 (unknown) | 126 | 6 (unknown) |
| Pfaar 2013 | Pollen | 94 | 24 (unknown) | 175 | 57 (unknown) |
| Colás | Pollen | 19 | 6 (mild) | 41 | 13 (mild) |
| Garcia-Robaina | Mites | 32 | 3 (mild)  29 (moderate) | 32 | 3 (mild)  29 (moderate) |
| Ameal | Mites | 26 | 26 (mild/moderate) | 29 | 26 (mild/moderate) |
