# Supplementary material for: Subcutaneous immunotherapy with depigmented-polymerized allergen extracts: a systematic review and meta-analysis
Source: Clin Transl Allergy. 2019 Jun 5;9:29. doi: 10.1186/s13601-019-0268-5 (PMC6549305; doi:10.1186/s13601-019-0268-5)
Supplement: Supplementary file 3 — Additional file 3. Articles selected for systematic review. Tabular display of all articles selected for systematic review. Articles selected for analysis are highlighted in bold. The following sources were used: Embase (*1), MEDLINE (*2), Cochrane (*3), LILACS (*4) and BIBLIOGRAPHY REVIEW FROM OTHER SELECTED ARTICLES (*5). [file 13601_2019_268_MOESM3_ESM.docx]

**Additional file 3. Articles selected in the systematic review.**

| **Source number** | | | | **ARTICLES** | **INCLUSION/**  **EXCLUSION** |
| --- | --- | --- | --- | --- | --- |
| ***1** | ***2** | ***3** | ***4** |  | **In meta-analysis** |
| **1** |  |  |  | Iraola V.M. Gallego M.T. Morales M. Taules M. Carnés J.  In vitro evaluation of the efficacy and safety of a depigmented-polymerized extract of cat epithelia  Journal of Allergy and Clinical Immunology (2016) 137:2 SUPPL. 1 (AB161). | Excluded after abstract review |
| **2** |  |  |  | Morais-Almeida M. Arêde C. Sampaio G. Borrego L.M.  Ultrarush schedule of subcutaneous immunotherapy with modified allergen extracts is safe in paediatric age  Asia Pacific Allergy (2016) 6:1 (35-42). Date of Publication: 2016 | Inappropriate study design |
| **3** |  |  |  | Sager A.  Aluminum pharmacokinetics in serum and urine following 1-year high dose SCIT with a depigmented polymerized birch pollen extract  Allergy: European Journal of Allergy and Clinical Immunology (2015) 70 SUPPL. 101 (230). | Excluded after abstract review |
| **4** |  | **1** |  | Sanchez Alonso A , Carretero Anibarro P , Mencia Bartolome J , Cerecedo Carballo I , Minano Oyarzabal A , Gomez Sanchez MJ and Levitch Meran R. A double-blind, placebo-controlled study of allergen immunotherapy with depigmented -polymerised *Phleum pratense* pollen extract in allergic rhinoconjunctivitis, a placebo-based analysis.  Allergy: European Journal of Allergy and Clinical Immunology, 2015, 70, 260 Publication Year: 2015 | Conference abstract. the final data were not published in a peer-reviewed journal |
| **5** |  |  |  | Carnés J. Gallego M.T. Morales M. López Matas M.Á. Moya R. Leonor J.R. Iraola V.  Biochemical and immunological characterization of a depigmentedpolymerized extract of cat epithelia  Allergy: European Journal of Allergy and Clinical Immunology (2015) 70 SUPPL. 101 (467). | Excluded after abstract review |
| **6** |  |  |  | Carnés J. Morales M. Moya R. López Matas M.A. Aranda T. Rojas B. Martinez B. Leonor J.R. Iraola V.M. Gallego M.T.  Characterization of depigmented-polymerized pollen extracts for allergen immunotherapy: Presence of relevant allergens and molecular size consistency.  Journal of Allergy and Clinical Immunology (2015) 135:2 SUPPL. 1 (AB59). | Excluded after abstract review |
| **7** | **1** |  |  | Pfaar O, Sager A, Robinson DS. Safety and effect on reported symptoms of depigmented polymerized allergen immunotherapy: a retrospective study of 2927 paediatric patients. Pediatr Allergy Immunol. 2015 May;26(3):280-6. | Excluded after abstract review |
| **8** |  |  |  | Iraola V.M. Leonor J.R. Morales M. Moya R. Lopez-Matas M.A. Gallego M.T. Carnés J.  Immunogenicity and IGE blocking capacity of a mixture of depigmented and  chemically modified allergens from different homologous groups  Journal of Allergy and Clinical Immunology (2014) 133:2 SUPPL. 1 (AB46). | Excluded after abstract review |
| **9** | **2** |  |  | Cardona R, Lopez E, Beltrán J, Sánchez J. Safety of immunotherapy in patients with rhinoconjunctivitis, asthma or atopic dermatitis using an ultra-rush buildup. A retrospective study. Allergol Immunopathol (Madr). 2014 Mar-Apr;42(2):90-5. doi: 10.1016/j.aller.2012.07.005. Epub 2012 Dec 20. PubMed PMID: 23265265. | Inappropriate study design |
| **10** |  |  |  | López-Matas M.A. Gallego M. Iraola V. Robinson D. Carnés J.  Depigmented allergoids reveal new epitopes with capacity to induce IgG  blocking antibodies  BioMed Research International (2013) 2013 Article Number: 284615. | Excluded after abstract review |
| **11** | **3** | **2** |  | **Pfaar O, Biedermann T, Klimek L, Sager A, Robinson DS. Depigmented-polymerized mixed grass/birch pollen extract immunotherapy is effective in polysensitized patients. Allergy. 2013 Oct;68(10):1306-13. doi: 10.1111/all.12219. Epub 2013 Sep 2. PubMed PMID: 23991896.(11)** | **Included (1)** |
| **12** |  |  |  | Arêde C. Sampaio G. Miguel Borrego L. Morais-Almeida M.  Ultrarush specific's immunotherapy safety using modified extracts in  pediatric age.  Revista Portuguesa de Imunoalergologia (2013) 21:2 (91-102). | Inappropriate study design |
| **13** |  |  |  | Carnes J. Lopez-Matas M.A. Gallego M.T. Aranda T. Morales M. Leonor J.R. Iraola V.M.  Depigmented-polymerized and native extracts of birch pollen exhibit  different IgG epitopes specific to bet v 1 and bet v 2  Journal of Allergy and Clinical Immunology (2013) 131:2 SUPPL. 1 (AB110) | Excluded after abstract review |
| **14** |  |  |  | Iraola V. Leonor J.R. Morales M. Lopez-Matas M.A. Gallego M.T. Carnes J.  Enzymatic activity of grass native allergenic extracts is eliminated in  depigmented allergoids.  Journal of Allergy and Clinical Immunology (2013) 131:2 SUPPL. 1 (AB110). | Excluded after abstract review |
| **15** |  |  |  | Sanchez J. Juliana B. Lopez E. Cardona R.  Adverse reactions to subcutaneous allergen-specific immunotherapy in patients with atopic dermatitis with a rush buildup  Allergy: European Journal of Allergy and Clinical Immunology (2012) 67 SUPPL. 96 (133). | Excluded after abstract review |
| **16** |  |  |  | Nieto A. Calderon R. Pina R. Uixera S. Mazon A. Cortell I. López J. Perea E.  Meta-analysis on immunotherapy with depigmented glutaraldehyde-polymerized extracts.  Allergy: European Journal of Allergy and Clinical Immunology (2012) 67 SUPPL. 96 (614). | Review article; no original data |
| **17** | **4** | **3** |  | Novak N, Bieber T, Hoffmann M, Fölster-Holst R, Homey B, Werfel T, Sager A, Zuberbier T. Efficacy and safety of subcutaneous allergen-specific immunotherapy with depigmented polymerized mite extract in atopic dermatitis. J Allergy Clin Immunol. 2012 Oct;130(4):925-31.e4. doi: 10.1016/j.jaci.2012.08.004. Epub 2012 Sep 1. | Inappropriate disease |
| **18** |  |  |  | Iraola V. Gallego M.T. López-Matas M.A. Morales M. Bel I. García N. Carnés J.  Immunogenicity of *Phleum pratense* depigmented allergoid vaccines:  Experimental study in rabbits  Journal of Investigational Allergology and Clinical Immunology (2012) 22:1 (35-40). | Excluded after abstract review |
| **19** | **5** | **4** |  | **Pfaar O, Urry Z, Robinson DS, Sager A, Richards D, Hawrylowicz CM, Bräutigam M, Klimek L. A randomized placebo-controlled trial of rush preseasonal depigmented polymerized grass pollen immunotherapy. Allergy. 2012 Feb;67(2):272-9. doi: 10.1111/j.1398-9995.2011.02736.x. Epub 2011 Nov 23. PubMed PMID: 22107266.(10)** | **Included (2)** |
| **20** |  |  |  | Gruber M. Amon U.  Ultra-rush specific immune therapy with depigmented and polymerized allergen extracts is effective and safe in patients with severe SAR highly sensitized against pollen allergens  World Allergy Organization Journal (2012) 5 SUPPL. 2 (S18). | Inappropriate study design |
| **21** |  |  |  | Sager A. Worm M.  Dose optimizing study of a depigmented polymerized allergen extract of birch pollen by means of conjunctival provocation test.  World Allergy Organization Journal (2012) 5 SUPPL. 2 (S18). Date of | Outcomes were not total symptoms or medication scores |
| **22** |  |  |  | Sager A. Biedermann T. Pfaar O.  Comparison of efficacy and safety of a depigmented polymerized allergen extract of grass and birch with placebo in patients with type-1 allergic rhinoconjunctivitis  World Allergy Organization Journal (2012) 5 SUPPL. 2 (S20). | Conference abstract; final data were published in the included study no. 1 |
| **23** |  |  |  | Abstracts of the 22nd World Allergy Congress  World Allergy Organization Journal (2012) 5 SUPPL. 2. | Excluded after abstract review |
| **24** |  |  |  | Iraola V. Gallego M.T. Aranda T. Lopez-Matas M.A. Morales M. Saez R. Marquez  C. Leonor J.R. Carnes J.  Inhibition of human IgE-allergen interaction by antibodies of rabbits  immunized with depigmented-polymerized allergen extracts.  Journal of Allergy and Clinical Immunology (2012) 129:2 SUPPL. 1 (AB115). | Excluded after abstract review |
| **25** |  | **5** |  | Pfaar O, Biedermann T, Sager A. Seasonal versus symptom-based evaluation of a depigmented grass-birch allergoid. Conference Abstract. World Allergy Organization journal. | Conference abstract: final data were published in the included study no. 1 |
| **26** |  |  |  | Carnes J. Gallego M.T. Taules M. Lopez-Matas M.A. Morales M. Saez R. Leonor J.R. Marquez C. Aranda T. Iraola V.  Comparison of the IgE interaction in depigmentedpolymerized and native  allergen extracts by surface plasmon resonance biosensor analysis  Journal of Allergy and Clinical Immunology (2012) 129:2 SUPPL. 1 (AB241). | Excluded after abstract review |
| **27** |  |  |  | Novak N. Zuberbier T. Sager A.  Efficacy and safety of a depigmented polymerised mite extract in patients suffering from atopic eczema with clinical relevant IgE-mediated sensitisation against house dust mites  Allergy: European Journal of Allergy and Clinical Immunology (2011) 66 SUPPL. 94 (103). | Inappropriate disease |
| **28** |  |  |  | Novak N. Thaci D. Hoffmann M. Fölster-Holst R. Biedermann T. Homey B. Schaekel K. Stefan J.A. Werfel T. Bieber T. Sager A. Zuberbier T.  Subcutaneous immunotherapy with a depigmented polymerized birch pollen extract - A new therapeutic option for patients with atopic dermatitis. International Archives of Allergy and Immunology (2011) 155:3 (252-256). | Inappropriate disease |
| **29** |  |  |  | Costa-Colomer J. Bosque M. Perea E. González M. Roca G. Valdesoiro L. Larramoma H. Peñas A. Asensio Ò. Garcia M.  Safety of rush immunotherapy with depigmented glutaraldehyde-modified dust mite extracts in children.  Allergy: European Journal of Allergy and Clinical Immunology (2011) 66 SUPPL. 94 (558-559). | Outcomes were not total symptoms or medication scores |
| **30** | **6** | **6** |  | **Pfaar O, Robinson DS, Sager A, Emuzyte R. Immunotherapy with depigmented-polymerized mixed tree pollen extract: a clinical trial and responder analysis. Allergy. 2010 Dec;65(12):1614-21. doi: 10.1111/j.1398-9995.2010.02413.x. PubMed PMID: 20645937.(9)** | **Included (3)** |
| **31** | **7** | **7** |  | **Höiby AS, Strand V, Robinson DS, Sager A, Rak S. Efficacy, safety, and immunological effects of a 2-year immunotherapy with Depigoid birch pollen extract: a randomized, double-blind, placebo-controlled study. Clin Exp Allergy. 2010 Jul;40(7):1062-70. doi: 10.1111/j.1365-2222.2010.03521.x. PubMed PMID: 20642579.(12)** | **Included (4)** |
| **32** |  |  |  | Ludger K. Matthias B. Sager A. Pfaar O.  Ultra-short preseasonal immunotherapy of depiquick grass pollen extract compared to placebo in patients with type-I allergic rhinoconjunctivitis  Allergy: European Journal of Allergy and Clinical Immunology (2010) 65 SUPPL. 92 (261-262). Date of Publication: June 2010 | Conference abstract; final data were published in the included study no. 2 |
| **33** | **8** |  |  | Pfaar O, Klimek L, Sager A, Bräutigam M. Safety of a depigmented, polymerized vaccine for the treatment of allergic rhinoconjunctivitis and allergic asthma. Am J Rhinol Allergy. 2010 May-Jun;24(3):220-5. doi: 10.2500/ajra.2010.24.3437. Epub  2010 Feb 17. PubMed PMID: 20167138. | Inappropriate study design |
| **34** | **9** | **8** |  | Brehler R, Klimek L, Pfaar O, Hauswald B, Worm M, Bieber T. Safety of a rush immunotherapy build-up schedule with depigmented polymerized allergen extracts. Allergy Asthma Proc. 2010 May-Jun;31(3):e31-8. doi: 10.2500/aap.2010.31.3334.  PubMed PMID: 20615317. | Inappropriate treatment comparators |
| **35** | **10** |  |  | Klimek L, Thorn C, Pfaar O. [Specific immunotherapy with depigmented allergoids]. HNO. 2010 Jan;58(1):51-6. doi: 10.1007/s00106-009-2006-0. German. PubMed PMID: 20011999. | Inappropriate study design |
| **36** | **11** |  |  | Himly M, Carnés J, Fernández-Caldas E, Briza P, Ferreira F. Characterization of allergoids. Arb Paul Ehrlich Inst Bundesinstitut Impfstoffe Biomed Arzneim Langen Hess. 2009;96:61-9; discussion 69-70. PubMed PMID: 20799446. | Excluded after abstract review |
| **37** |  |  |  | Biller H. Badorrek P. Hohlfeld J. Krug N. Sager A.  Efficacy of a rush immunotherapy with a depigmented polymerised extract of grass pollen assessed in an Environmental Challenge Chamber. Allergy: European Journal of Allergy and Clinical Immunology (2009) 64 SUPPL. 90 (345). | Outcomes were not total symptoms or medication scores |
| **38** |  |  |  | Iraola V. Carnés J. Badiola C. Robinson D. García Robaina J.  Immunological changes after treatment with a depigmented and polymerised  allergenic extract of *D. pteronyssinus* and *D. farinae*  Allergy: European Journal of Allergy and Clinical Immunology (2009) 64 SUPPL. 90 (358). | Outcomes were not total symptoms or medication scores |
| **39** |  | **9** |  | Sager A and Emuzyte R. [Comparison of efficacy and safety of a depigmented tree pollen extract with placebo in patients with type-I allergic rhinoconjunctivitis.](http://onlinelibrary.wiley.com/o/cochrane/clcentral/articles/523/CN-01029523/frame.html) Allergy: European Journal of Allergy and Clinical Immunology, 2009, 64, 344. | Conference abstract; final data were published in the included study no. 3 |
| **40** |  |  |  | Carnés J. Gallego M. Himly M. Iraola V. Briza P.  Physicochemical characterization of polymerized extracts in *Dermatophagoides pteronyssinus*.  Journal of Allergy and Clinical Immunology (2009) 123:2 SUPPL. 1 (S164). | Excluded after abstract review |
| **41** | **12** |  |  | Casanovas M, Martín R, Jiménez C, Caballero R, Fernández-Caldas E. Safety of immunotherapy with therapeutic vaccines containing depigmented and polymerized allergen extracts. Clin Exp Allergy. 2007 Mar;37(3):434-40. PubMed PMID: 17359393.(7) | Inappropriate study design |
| **42** | **13** | **10** |  | **García-Robaina JC, Sánchez I, de la Torre F, Fernández-Caldas E, Casanovas M. Successful management of mite-allergic asthma with modified extracts of *Dermatophagoides pteronyssinus* and *Dermatophagoides farinae* in a double-blind, placebo-controlled study. J Allergy Clin Immunol. 2006 Nov;118(5):1026-32. Epub 2006 Sep 18. PubMed PMID: 17088125.(16)** | **Included (5)** |
| **43** | **14** | **11** |  | Ibero M, Castillo MJ. Significant improvement of specific bronchial  hyperreactivity in asthmatic children after 4 months of treatment with a modified extract of *Dermatophagoides pteronyssinus*. J Investig Allergol Clin Immunol. 2006;16(3):194-202. PubMed PMID: 16784014. | Inappropriate study design |
| **44** | **15** | **12** |  | **Colás C, Monzón S, Venturini M, Lezaun A. Double-blind, placebo-controlled study with a modified therapeutic vaccine of *Salsola kali* (Russian thistle) administered through use of a cluster schedule. J Allergy Clin Immunol. 2006 Apr;117(4):810-6. Epub 2006 Feb 8. PubMed PMID: 16630938.(13)** | **Included (6)** |
| **45** | **16** |  |  | Casanovas M, Martín R, Jiménez C, Caballero R, Fernández-Caldas E. Safety of an ultra-rush immunotherapy build-up schedule with therapeutic bacines containing depigmented and polymerized allergen extracts. Int Arch Allergy Immunol. 2006;139(2):153-8. Epub 2005 Dec 22. PubMed PMID: 16374026. | Inappropriate study design |
| **46** |  |  |  | Álvarez-Cuesta E. Aragoneses-Gilsanz E. Martín-García C. Berges-Gimeno P. González-Mancebo E. Cuesta-Herranz J.  Erratum: Immunotherapy with depigmented glutaraldehyde-polymerized extracts:  Changes in quality of life (Clinical and Experimental Allergy (2005) 35, (572-578)) Clinical and Experimental Allergy (2005) 35:11 (1504). | Excluded after abstract review |
| **47** | **17** | **13** |  | Casanovas M, Sastre J, Fernández-Nieto M, Lluch M, Carnés J, Fernández-Caldas E. Double-blind study of tolerability and antibody production of unmodified and chemically modified allergen vaccines of *Phleum pratense*. Clin Exp Allergy. 2005 Oct;35(10):1377-83. Erratum in: Clin Exp Allergy. 2005 Dec;35(12):1651-2. PubMed PMID: 16238799. | Outcomes were not total symptoms or medication scores |
| **48** | **18** | **14** |  | Casanovas M, Fernández-Caldas E, Alamar R, Basomba A. Comparative study of tolerance between unmodified and high doses of chemically modified allergen vaccines of *Dermatophagoides pteronyssinus*. Int Arch Allergy Immunol. 2005 Jul;137(3):211-8. Epub 2005 Jun 9. PubMed PMID: 15956789. | Outcomes were not total symptoms or medication scores |
| **49** |  |  |  | Casanovas M. Gómez M.J. Carnés J. Fernández-Caldas E.  Skin test with native, depiqmented and glutaraldehyde polymerized allergen extracts. Journal of Investigational Allergology and Clinical Immunology (2005) 15:1 (30-36). | Excluded after abstract review |
| **50** | **19** | **15** |  | **Alvarez-Cuesta E, Aragoneses-Gilsanz E, Martín-Garcia C, Berges-Gimeno P, Gonzalez-Mancebo E, Cuesta-Herranz J. Immunotherapy with depigmented glutaraldehyde-polymerized extracts: changes in quality of life. Clin Exp Allergy. 2005 May;35(5):572-8. Erratum in: Clin Exp Allergy. 2005 Nov;35(11):1504. PubMed PMID: 15898977.(15)** | **Included (7)** |
| **51** | **20** | **16** |  | Branco Ferreira M, Spínola Santos A, Pereira Santos MC, Palma Carlos ML, Pereira Barbosa MA, Palma Carlos AG. Efficacy and safety of specific immunotherapy with a modified mite extract. Allergol Immunopathol (Madr). 2005 Mar-Apr;33(2):80-5. PubMed PMID: 15808114. | Inappropriate study design |
| **52** |  |  |  | Sager A, Renner, BG. Safety aspects and course of allergen-specific immunotherapy with a depigmented, polymerized allergen extract  (2004) Allergo Journal, 13 (6), pp. 386-391. | Inappropriate study design |
| **53** | **21** | **17** |  | Ferrer A, García-Sellés J. Significant improvement in symptoms, skin test, and specific bronchial reactivity after 6 months of treatment with a depigmented, polymerized extract of *Dermatophagoides pteronyssinus* and *D. farinae*. J Investig Allergol Clin Immunol. 2003;13(4):244-51. PubMed PMID: 14989113. | Inappropriate study design |
| **54** | **22** | **18** |  | Guerra F, Daza JC, Almeda E. Immunotherapy with a depigmented, polymerized vaccine of *Olea europaea* pollen allergens. Significantly reduces specific bronchial and skin test reactivity in sensitized patients after one year of treatment. J Investig Allergol Clin Immunol. 2003;13(2):108-17. PubMed PMID: 12968395. | Inappropriate study design |
| **55** | **23** | **19** |  | García-Sellés J, Pascual A, Funes E, Pagán JA, López JD, Negro JM, Hernández J. Clinical efficacy and safety of a depigmented and glutaraldehyde polymerized therapeutic vaccine of *Parietaria judaica*. Allergol Immunopathol (Madr). 2003 Mar-Apr;31(2):63-9. PubMed PMID: 12646120. | Inappropriate study design |
| **56** | **24** |  |  | Ferrer A, García-Sellés J. SIT with a depigmented, polymerized mite extract. Allergy. 2002 Aug;57(8):754-5. PubMed PMID: 12121200. | Outcomes were not total symptoms or medication scores |
| **57** |  |  |  | Casanovas M.  Allergens that are depigmented and polymerized with glutaraldeyde  Alergologia e Inmunologia Clinica (1999) 14:4 (242). | Excluded after abstract review |
|  |  | **20** |  | Gallego MT, Iraola V, Himly M, Robinson DS, Badiola C, García-Robaina JC, et al. Depigmented and polymerised house dust mite allergoid: allergen content, induction of IgG4 and clinical response. Int Arch Allergy Immunol. 2010;153(1):61-9. | Ooutcomes were not total symptoms or medication scores |
|  | **25** |  |  | Hernández N, Ibero M, Ridao M, Artigas R, Viñas M, Castillo MJ. Safety of specific immunotherapy using a depigmented and polymerised extract of *Dermatophagoides pteronyssinus* in children under five years of age. Allergol Immunopathol (Madr). 2011 Sep-Oct;39(5):267-70. doi: 10.1016/j.aller.2010.09.002.  Epub 2011 Feb 18. PubMed PMID: 21334128. | Inappropriate disease |
|  | **26** |  |  | g N, Thaci D, Hoffmann M, Fölster-Holst R, Biedermann T, Homey B, Schaekel K, Stefan JA, Werfel T, Bieber T, Sager A, Zuberbier T. Subcutaneous immunotherapy with a depigmented polymerized birch pollen extract--a new therapeutic option for patients with atopic dermatitis. Int Arch Allergy Immunol. 2011;155(3):252-6. doi: 10.1159/000320058. Epub 2011 Feb 2. PubMed PMID: 21293143. | Inappropriate disease |
|  | **27** |  |  | Casanovas M, Gómez MJ, Carnés J, Fernández-Caldas E. Skin tests with native, depigmented and glutaraldehyde polymerized allergen extracts. J Investig Allergol Clin Immunol. 2005;15(1):30-6. Erratum in: J Investig Allergol Clin Immunol.2005;15(3):233. PubMed PMID: 15864880. | Outcomes were not total symptoms or medication scores |
|  |  |  | **1** | Skrie, Victor Claudio. Allergen immunotherapy in under 5 children with asthma (Doctoral Thesis Abstract) Alerg. inmunol. clin; 31(1/2): 26-36, 2012. | Excluded after abstract review |
|  |  |  | **2** | Pereira, Celso; Botelho, Filomena; Tavares, Beatriz; Lourenço, Cândida; Baeta, Cristina; Palma - Carlos, AG; Lima, João;Chieira, Celso. Specific immunotherapy - in vivo dynamic and kinetic evaluation of a therapeutic extract in allergic patients. Rev. bras. alergia imunopatol; 28(1): 9-19, jan.-fev. 2005. | Excluded after abstract review |
|  |  |  | **3** | Gómez Tello, Héctor; Paz Martínez, David; Galindo García, José Arturo; Jiménez Montiel, José Antonio; Melgarejo Bibiano, Lizbeth; Toriz Martínez, Eduardo. Allergen-specific immunotherapy: causes of abandon during the first year after initialization at the Universitary Hospital of Puebla Alergia inmunol. pediátr; 5(2): 44-7, mar.-abr. 1996. | Excluded after abstract review |
|  |  |  | **4** | Romero Vega, Lorenzo; Huerta López, José G; Pedroza Meléndez, Alvaro; Velasco Ortiz, Romelia; de Martínez, Cristina S. Bronchial and skin hyperreactivity postimmunotherapy in children  Alergia inmunol. pediátr; 5(2): 48-53, mar.-abr. 1996. | Excluded after abstract review |
|  |  |  | **5** | Crisci, Carlos D; Ardusso, Ledit. Utility of IgE totality serie determination a control immunotherapy. Arch. argent. alerg. inmunol. clín; 21(1): 7-17, mar. 1990. Ilus | Excluded after abstract review |
|  |  |  | **6** | Espinoza Morales, Sylvia Marcela; Bolaños Ancona, Jorge Carlos; Miranda Feria, Alfonso Javier. Clinic evaluation of the 2 per cent cromoglycate sodium chomoglycate nasal solution in the allergic rinitis Alergia; 40(1): 3-9, ene.-feb. 1993. | Excluded after abstract review |
|  |  |  | **7** | Yang, Ariana C; Arruda, Luisa K; Kokron, Cristina M; Galvão, Clóvis E. S; Kalils, Jorge; Castro, Fábio.Cross-reactivity between dust mite and shrimp: what is the effect of immunotherapy?  Rev. bras. alergia imunopatol; 33(1): 14-22, jan.-fev. 2010. | Excluded after abstract review |
|  |  |  | **8** | Rodrigues, Adriana T; Fernandes, Fátima R; Aun, Wilson T; Melo, João F. de; Carvalho, Andréa P. E. de; Silva, Bárbara G. da.  Clinical characteristics of patients with difficult asthma  Rev. bras. alergia imunopatol; 30(2): 56-61, mar.-abr. 2007. Tab+ | Excluded after abstract review |
|  |  | **21** |  | Novak N, Bieber T, Hoffmann M, Fölster-Holst R, Homey B, Werfel T, Sager A, Zuberbier T. Efficacy and safety of subcutaneous allergen-specific immunotherapy with depigmented polymerized mite extract in atopic dermatitis. J Allergy Clin Immunol. 2012 Oct;130(4):925-31.e4. doi: 10.1016/j.jaci.2012.08.004. Epub 2012 Sep 1. | It is the same reference as Cochrane no. 3; duplicate |
| ***5** | | | | **Ameal A, Vega-Chicote JM, Fernández S, Miranda A, Carmona MJ, Rondón MC, et al. Double-blind and placebo-controlled study to assess efficacy and safety of a modified allergen extract of *Dermatophagoides pteronyssinus* in allergic asthma. Allergy. 2005;60(9):1178-83.(14)** | **Included (8)** |
